# Accretionary pedogenesis and Holocene climate evolution in black soil region of northeast China: Evidence from a sedimentary profile

**DOI:** 10.1371/journal.pone.0348110

**Published:** 2026-09-21

**Authors:** Yangyang Chen, Ke Yang, Fubing He, Junbo Yu, Zhiwei Yang, Shaozhong Qiao, Jiayu Wang, Xinyi Wang, Jiacheng Liu, Xue Liu, Chenchen Wang

**Affiliations:** 1 Harbin Natural Resources Survey, China Geological Survey, Harbin, Heilongjiang, China; 2 Northeast Geological S&T Innovation Center of China Geological Survey, Shenyang, Liaoning, China; 3 Observation and Research Station of Earth Critical Zone in Black Soil, Ministry of Natural Resources, Harbin, China; 4 Institute of Geophysical and Geochemical Exploration, Chinese Academy of Geological Sciences, Langfang, Hebei, China; 5 Beijing Institute of Geological Survey, Beijing, China; Institute of Earth and Environment, Chinese Academy of Sciences, CHINA

## Abstract

The black soil region of the Songnen Plain, one of the world’s three major black soil belts, is critical for China’s grain security, yet the formation mechanism of its thick, organic-rich soils remains insufficiently quantified. Here, we investigate the ZYHPM01 profile in the eastern Songnen Plain using grain-size end-member analysis (EMA), multi-proxy geochemical tracers, and coarse-grained quartz optically stimulated luminescence (OSL) dating. The EMA identifies three sedimentary components: EM1 represents distal background dust transported via high-altitude suspension; EM2 and EM3 indicate proximal dust inputs associated with the winter monsoon. A Bayesian age-depth model constructed from five OSL ages establishes the chronostratigraphic framework, dividing the Holocene evolution into five stages. Following rapid accumulation in the Early Holocene (12.07–10.31 ka BP), the region experienced a cooling episode during the Early-to-Middle Holocene transition, broadly consistent with high-latitude cooling events such as the 8.2 ka event. The Mid-Holocene Climatic Optimum (6.72–5.11 ka BP) established a warm-humid environment, with elevated Chemical Index of Alteration (CIA) values (avg. ~ 64.4) and minimum Sr/Cu ratios (~6.88) indicating moderate chemical weathering and leaching; this interval served as the primary environmental window for black soil initiation. An abrupt shift to cold-dry conditions in the Late Holocene, marked by enhanced coarse-grained dust inputs driven by a strengthened winter monsoon, terminated this optimum. We propose a formation-preservation hypothesis: Mid-Holocene productivity formed the organic-rich substrate, while subsequent climatic cooling likely inhibited microbial decomposition and restricted chemical leaching, thereby favoring preservation of the accumulated organic carbon. These findings suggest that the black soil at ZYHPM01 represents Mid-Holocene productivity preserved by Late Holocene climatic cooling, offering new insights into critical zone evolution at monsoon margins.

## 1. Introduction

The black soil region of the eastern Songnen Plain is a cornerstone of China’s grain security and a vital component of the global food system [1,2]. Although renowned for its deep humus layer and exceptional fertility, this soil resource faces severe degradation under anthropogenic pressure [3–6]. Developing effective conservation strategies requires a thorough understanding of the natural processes and timescales governing black soil formation. Unlike soils developed on stable bedrock, the black soil in this region is a product of accretionary pedogenesis, whereby pedogenesis proceeds simultaneously with continuous aeolian dust deposition [7,8]. Consequently, the genesis of black soil is recorded not only in its chemical weathering signatures but also in the physical dynamics of sediment accumulation driven by the East Asian Monsoon.

Previous studies have established a foundational understanding of the timing and provenance of black soil in the Songnen Plain. The initiation of black soil development is generally traced to the early-to-mid Holocene, no later than 8.5 ka BP [9,10]. Its parent material is broadly recognized as multi-source, encompassing distal aeolian loess [11,12], alluvial deposits, and in situ residual materials [1], among which aeolian sediments are a key component. These materials are derived from a complex mixture of long-distance input from upwind arid regions (e.g., Gobi Desert, Mongolian Plateau) and proximal contributions from regional sandy lands such as the Horqin and Songnen fields [13–17]. During the Holocene, fluctuations in the East Asian Monsoon and the Westerlies have been identified as the primary drivers of wind regimes, dust transport pathways, and vegetation dynamics [18,19].

Despite this progress, reconstructing the evolutionary history of black soil remains challenging. First, disentangling the complex signals of aeolian deposition and pedogenic processes requires integrated multi-proxy approaches. Although geochemical proxies such as the Chemical Index of Alteration (CIA) and the Rb/Sr ratio are widely used as indicators of chemical weathering intensity, their interpretation in dynamic aeolian environments requires careful consideration of sediment provenance variations and post-depositional processes. Second, establishing a robust chronology is complicated by intense pedoturbation, which in black soils often compromises both bulk organic dating and fine-grained luminescence dating [20]. Consequently, the mechanisms governing the interplay between sediment accumulation rates and organic matter preservation remain insufficiently quantified.

This study addresses these challenges through an integrated investigation of the ZYHPM01 profile in the eastern Songnen Plain. We employed grain-size end-member analysis (EMA) [21–23] to quantitatively distinguish distal from proximal sedimentary components, and coarse-grained quartz (125–180 µm) for optically stimulated luminescence (OSL) dating to minimize the effects of post-depositional mixing of finer fractions. A Bayesian age-depth model was then applied to construct a robust chronostratigraphic framework. Building on the accretionary pedogenesis concept [7,8], we test the hypothesis that Holocene monsoon variability controlled black soil development through two coupled pathways: winter-monsoon intensity set the flux and grain-size of aeolian inputs and hence the pace of accretion, whereas summer-monsoon warm-humid intervals drove chemical weathering and biological productivity. If correct, the black soil should record a Mid-Holocene formation phase followed by a Late Holocene phase in which cooling favored preservation over production, detectable as a decoupling between weathering proxies and TOC. Accordingly, this study aims to: (1) reconstruct the Holocene sedimentary and climatic history of the eastern Songnen Plain; (2) characterize the relationship between aeolian deposition dynamics and chemical weathering intensity; and (3) test this hypothesis and refine a model of black soil formation and preservation. These findings contribute to a deeper understanding of black soil genesis in the Songnen Plain and its response to Holocene climate change, with implications for the sustainable management of this critical soil resource.

## 2. Materials and methods

### 2.1. Study region

**Fig 1:**
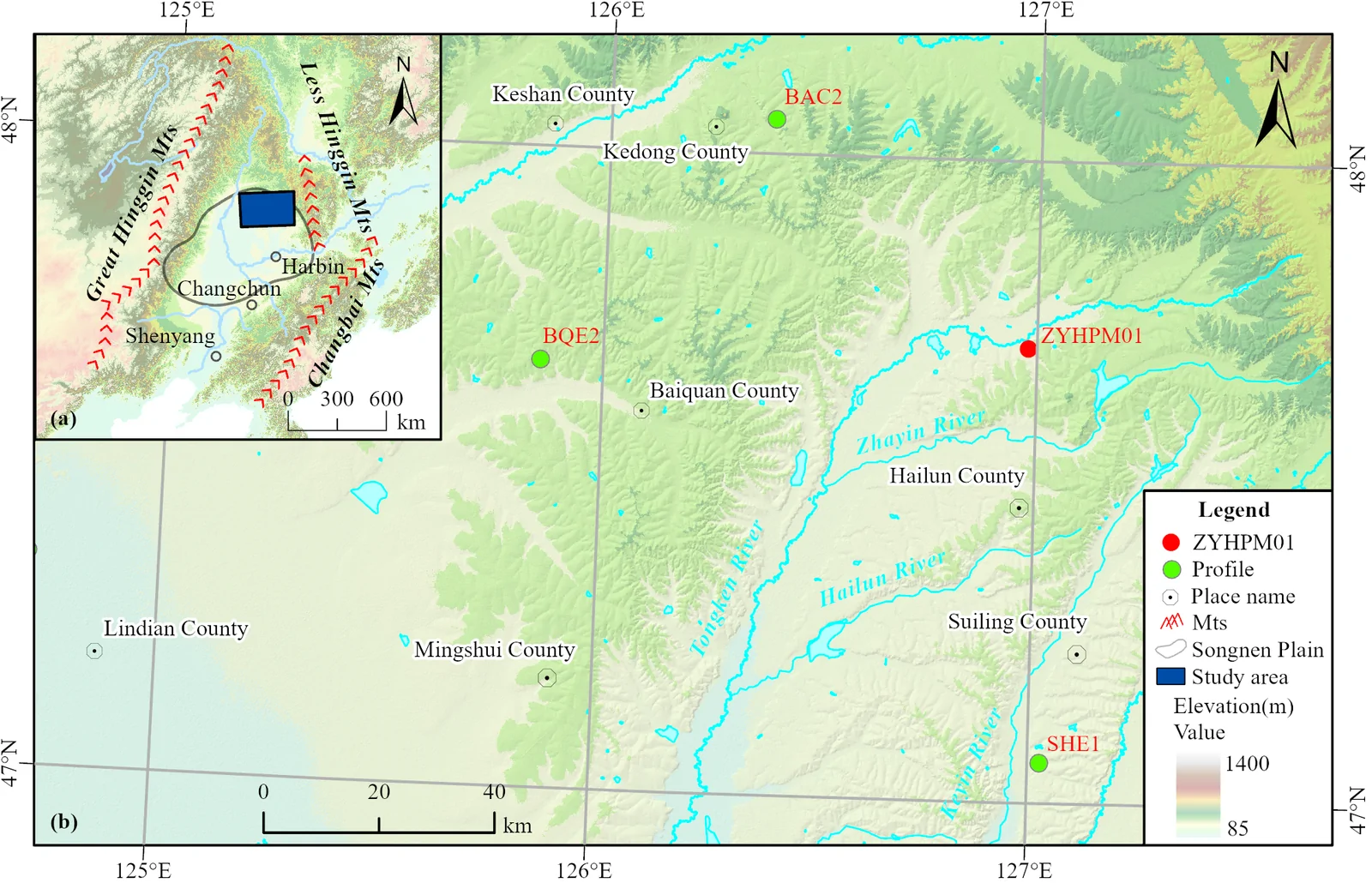
Location of the study area. (a) A schematic map showing the Songnen Plain in Northeast China and the location of the study area on its eastern side. (b) Topographic map of the study area showing the location of the ZYHPM01 profile. Base map elevation data from the Shuttle Radar Topography Mission (SRTM, doi:https://doi.org/10.5067/MEaSUREs/SRTM/SRTMGL1.003).

The Songnen Plain (Fig 1) is located in the northeastern region of China, bordered by the Greater Khingan Mountains to the west, the Lesser Khingan Mountains to the north, the Changbai Mountains to the east, and the Liaohe Plain to the south. The terrain slopes gently from the higher elevations in the north to lower elevations in the south, transitioning from peripheral mountainous areas to a central plain with a roughly diamond-shaped planform [24,25]. This plain formed on the tectonic foundation of the Mesozoic Songliao Basin and was subsequently shaped by aeolian activity, fluvial action, and lacustrine deposition during the Quaternary. The surface is characterized by thick accumulations of loosely consolidated Quaternary sediments [15]. The study area has a temperate continental monsoon climate with distinct seasons and synchronous rainfall and warm seasons. Cold, prolonged winters and dry, windy springs provide climatic conditions conducive to both aeolian dust transport and black soil development [26].

**Fig 2:**
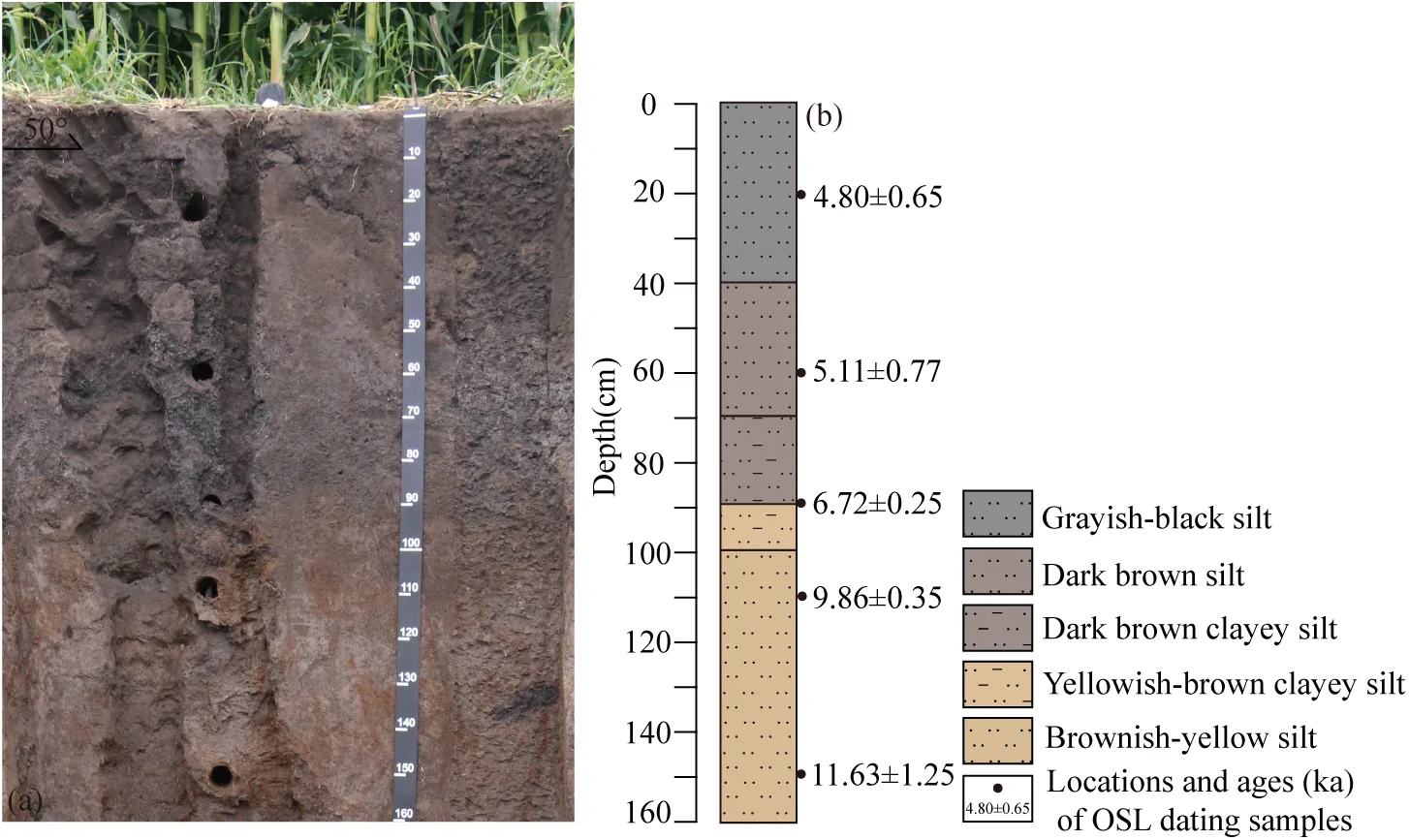
The ZYHPM01 profile. (a) Field photograph, taken by the authors, showing the exposed profile (1.6 m depth, orientation NE50°), with lithological boundaries and OSL sampling locations marked. (b) Schematic diagram showing the five lithological layers, OSL sampling depths (0.2, 0.6, 0.9, 1.1, and 1.5 m).

The ZYHPM01 profile (126.979304°E, 47.707992°N; elevation ~218.0 m) is located on a stable terrace crest approximately 1.3 km northeast of Dongqijiatun, Zhayinhe Township, Hailun City. The site lies within the undulating black soil region of the eastern Songnen Plain, a typical concentration zone of black soil [11]. The modern surface vegetation is cropland, with low natural vegetation cover. The profile, exposed on a northeast-facing slope (50°), extends to a depth of 1.6 m (Fig 2). No specific permits were required for the field sampling described in this study, as the sampling site is located on publicly accessible agricultural land and does not involve protected species or habitats. The field work was conducted in accordance with the regulations of the China Geological Survey.

### 2.2. OSL dating

After cleaning the profile, five OSL dating samples were collected at depths of 0.2, 0.6, 0.9, 1.1, and 1.5 m (Fig 2). Sampling was conducted under dark conditions. Stainless steel tubes (~5 cm diameter, 20 cm length) with one end sealed by a black plastic bag were hammered vertically into the freshly exposed profile face to obtain undisturbed sediment samples. Each sample weighed approximately 500 g. Both ends of the tubes were sealed with opaque covers and wrapped with black tape to prevent light exposure and moisture loss.

OSL sample pretreatment and measurement were conducted in the Luminescence Dating Laboratory, Jilin University. All procedures were performed in a darkroom under subdued red light. Carbonates and organic matter were removed with 10% HCl and 30% H_2_O_2_, respectively. Coarse-grained quartz (125–180 µm) was extracted for dating, as this fraction is less susceptible to post-depositional translocation than fine silt. The quartz fraction was etched with 40% HF for 40 min to remove the alpha-irradiated outer rind and eliminate feldspar contamination, followed by HCl rinsing. Quartz purity was verified by infrared stimulation (IR test). Equivalent dose (De) was determined using the single-aliquot regenerative-dose (SAR) protocol [27] on a Risø TL/OSL-DA-20 reader with blue-LED excitation (470 ± 30 nm) and a Hoya U-340 detection filter, at a preheat of 220 °C for 10 s and a measurement of 125 °C. Environmental dose rates were calculated from U, Th, and K concentrations measured by ICP-MS/ICP-OES. Cosmic dose rates were estimated following Prescott et al. [28], accounting for burial depth, altitude, and geographic location. Ages were calculated following Aitken and Smith [29]. Statistical treatment of De distributions to address partial bleaching and pedoturbation is described in Section 3.2. A Bayesian age-depth model was constructed using the Bacon package [30] in R to integrate the OSL ages into a continuous chronostratigraphic framework with quantified uncertainties.

### 2.3. Grain size measurement

Sixteen sediment samples were collected at 0.1 m intervals from the base to the top of the profile (Fig 2). Each sample was air-dried, and approximately 0.5 g was treated with 10% H_2_O_2_ to remove organic matter and 10% HCl to dissolve carbonates. After complete reaction, samples were repeatedly centrifuged and rinsed with deionized water to neutral pH. A 0.05 mol/L (NaPO_3_)_6_ dispersing agent was added, and the suspension was sonicated for 10 min to ensure complete particle dispersion. Grain-size measurements were performed at the Institute of Geophysical and Geochemical Exploration, Chinese Academy of Geological Sciences, using a Mastersizer 2000 laser diffractometer (Malvern Instruments, UK; measurement range 0.01–2000 µm; repeatability < 1%). As a quality check, 5% of the samples were randomly selected for duplicate analysis, all of which yielded consistent results. Grain-size fractions were classified according to the Udden-Wentworth scale, and statistical parameters (sorting, skewness, kurtosis) were calculated following Folk and Ward [31].

### 2.4. Chemical analysis

Sixteen samples, corresponding to the grain-size sampling horizons, were analyzed for elemental composition at the Harbin Natural Resources Survey. Samples were oven-dried at 45 °C to constant weight and ground to < 200 mesh using an agate mortar. Major elements were determined by X-ray fluorescence spectrometry (XRF; PANalytical Axios Max). Trace and rare earth elements (REE) were measured by inductively coupled plasma mass spectrometry (ICP-MS; Thermo Fisher Scientific Xseries 2) following digestion with HF-HNO_3_-HClO_4_ under high pressure. Total organic carbon (TOC) was determined by potassium dichromate oxidation (K_2_Cr_2_O_7_-H_2_SO^4^) with external heating. Quality assurance and quality control (QA/QC) procedures included: (1) analysis of certified reference materials (GSS series) alongside each batch, with recoveries of 95–105% for major elements and 90–110% for trace elements and TOC; (2) no fewer than two method blanks were processed alongside the samples in each batch. Blank values for all elements were below the corresponding instrumental detection limits, indicating that no detectable contamination was introduced during sample preparation and analysis; and (3) four duplicate samples were randomly inserted (replicate ratio ≥ 30%). The relative percent difference (RPD) was < 5% for major elements, < 10% for trace elements, and < 10% for TOC. The analytical methods and detection limits are summarized in Table 1. Full QA/QC details follow the protocols described in Li et al. [32].

**Table 1:** Laboratory analytical methods and detection limits.

Element	Unit	Analytical Methods	Detection Limit	Digestion Method
SiO_2_	%	XRF	0.095	Pressed powder pellets
Al_2_O_3_	%	XRF	0.045	Pressed powder pellets
TFe_2_O_3_	%	XRF	0.049	Pressed powder pellets
MgO	%	XRF	0.02	Pressed powder pellets
CaO	%	XRF	0.046	Pressed powder pellets
Na_2_O	%	XRF	0.03	Pressed powder pellets
K_2_O	%	XRF	0.01	Pressed powder pellets
Ba	μg/g	ICP-MS	8.53	HF + HCl + HNO_3_ + HClO_4_
Cu	μg/g	ICP-OES	0.85	HF + HCl + HNO_3_ + HClO_4_
Rb	μg/g	XRF	8	Pressed powder pellets
Sc	μg/g	ICP-OES	0.7	HF + HCl + HNO_3_ + HClO_4_
Sr	μg/g	ICP-MS	1.47	HF + HCl + HNO_3_ + HClO_4_
Th	μg/g	ICP-MS	0.07	HF + HCl + HNO_3_ + HClO_4_
La	μg/g	ICP-MS	1.13	HF + HCl + HNO_3_ + HClO_4_
Ce	μg/g	ICP-MS	0.7	HF + HCl + HNO_3_ + HClO_4_
Pr	μg/g	ICP-MS	0.01	HF + HCl + HNO_3_ + HClO_4_
Nd	μg/g	ICP-MS	0.05	HF + HCl + HNO_3_ + HClO_4_
Sm	μg/g	ICP-MS	0.02	HF + HCl + HNO_3_ + HClO_4_
Eu	μg/g	ICP-MS	0.01	HF + HCl + HNO_3_ + HClO_4_
Gd	μg/g	ICP-MS	0.05	HF + HCl + HNO_3_ + HClO_4_
Tb	μg/g	ICP-MS	0.03	HF + HCl + HNO_3_ + HClO_4_
Dy	μg/g	ICP-MS	0.02	HF + HCl + HNO_3_ + HClO_4_
Ho	μg/g	ICP-MS	0.03	HF + HCl + HNO_3_ + HClO_4_
Er	μg/g	ICP-MS	0.01	HF + HCl + HNO_3_ + HClO_4_
Tm	μg/g	ICP-MS	0.03	HF + HCl + HNO_3_ + HClO_4_
Yb	μg/g	ICP-MS	0.01	HF + HCl + HNO_3_ + HClO_4_
Lu	μg/g	ICP-MS	0.02	HF + HCl + HNO_3_ + HClO_4_
TOC	%	Vol	0.1	K_2_Cr_2_O_7_-H_2_SO_4_

### 2.5. Grain size EMA

Grain-size end-member modeling was performed on all 16 samples (100 size classes per sample) using the AnalySize package (v. 1.2.2) [33] in MATLAB R2023a. Following the theoretical framework of Prins et al. [34] and Weltje [35], non-negative least-squares and linear-programming algorithms were used to decompose the grain-size distributions into end members (EMs) with fixed distributions and variable contributions per sample. A three-EM model was adopted, as it yielded the highest coefficient of determination (R^2^ > 0.95) while maintaining geologically meaningful components; models with fewer EMs underfit the data, whereas those with more introduced statistical noise without improving interpretation. Although pedoturbation may partially blur EM signatures, the systematic stratigraphic trends in EM contributions indicate that primary depositional signals are largely preserved.

### 2.6. CIA calculation

The CIA was calculated from major-element data to evaluate the intensity of chemical weathering:


$$\begin{array}{c} {CIA=[Al_{2}O_{3}/(Al_{2}O_{3}+CaO}^{*}+Na_{2}O+K_{2}O)]\times 100 \end{array}$$ (1)

where all oxides are in molar proportions, and CaO* represents the CaO hosted in silicate minerals [36]. Because bulk CaO includes contributions from carbonate and phosphate minerals, CaO* was corrected following McLennan et al. [37]: when molar CaO ≤ molar Na_2_O, CaO* = CaO; when molar CaO > molar Na_2_O, CaO* = Na_2_O.

## 3. Results

### 3.1. Lithology characteristics

The ZYHPM01 profile is divided into five lithological layers based on field-observable characteristics (color, texture, and structure; Fig 2). From top to bottom:

Layer 1, 0–0.4 m, black soil layer; dark gray-brown silt with a loose, crumb structure; abundant organic matter and plant roots.
Layer 2, 0.4–0.7 m, black soil layer; blackish-brown sandy silt with a well-developed granular to subangular blocky structure; sparse plant roots.
Layer 3, 0.7–0.9 m, black soil layer, blackish-brown clayey silt with a well-developed granular structure; occasional plant fragments.
Layer 4, 0.9–1.0 m, yellowish-brown clayey silt, relatively homogeneous, with minor rust-colored mottles and iron-manganese nodules.
Layer 5, 1.0–1.6 m, yellowish-brown silt, slightly coarser texture, with localized lenses of fine sand and occasional calcareous nodules or pseudo-mycelia in the lower part.

Layers 1–3 constitute the black soil horizon, distinguished from the underlying yellowish-brown layers by their dark coloration (Munsell value ≤ 3), elevated TOC (>1.2%), well-developed soil structure, and homogeneous texture. The subdivision of the black soil is based on textural differences. It should be noted that these lithological layers are defined on macroscopic field criteria and do not correspond one-to-one with the evolutionary Stages (I–V) defined in Section 3.2 on the basis of integrated chronological and multi-proxy trends; this decoupling reflects the gradational nature of soil boundaries in accretionary pedogenic settings.

### 3.2. Chronology and sedimentation rate

**Fig 3:**
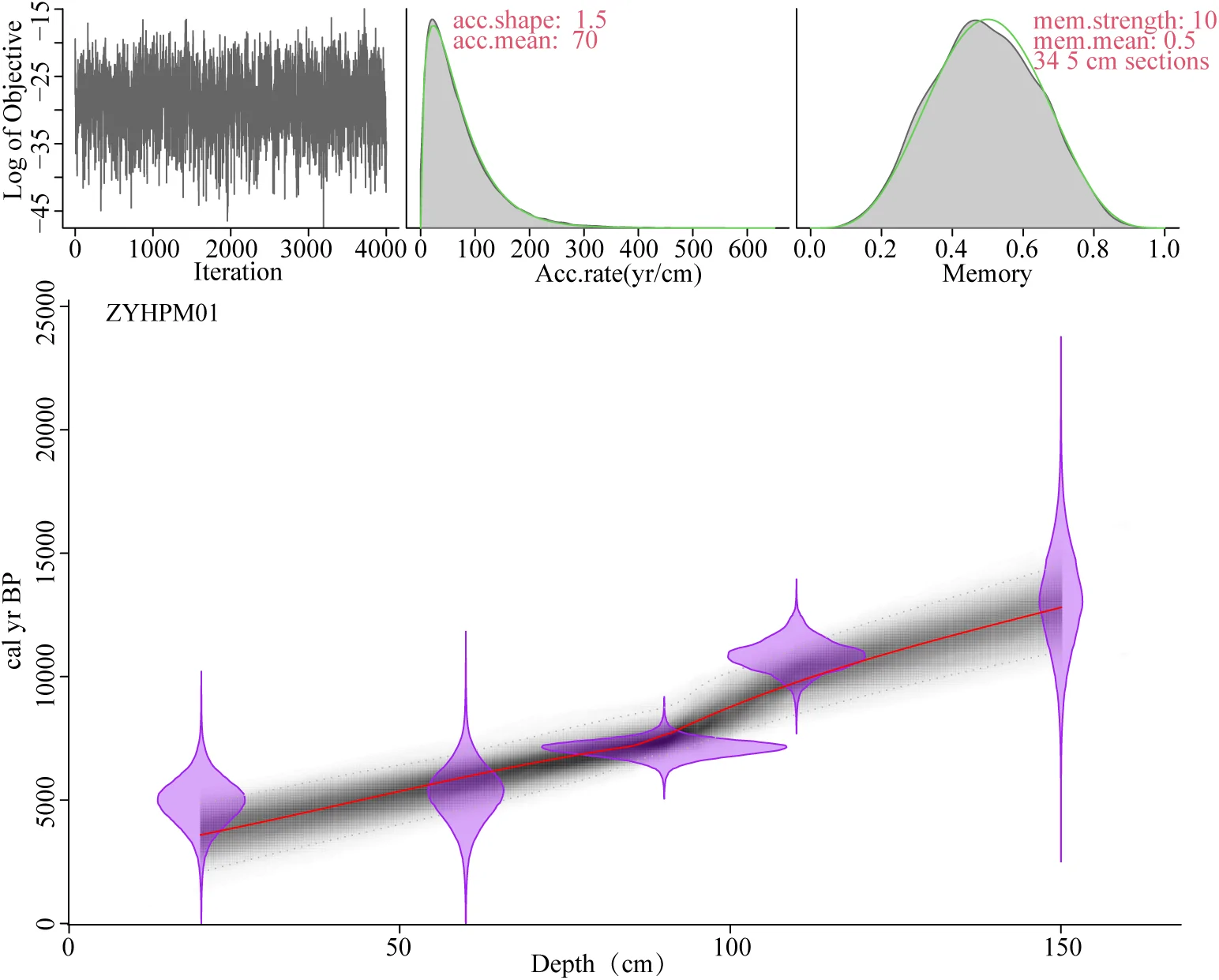
Bayesian age–depth model of the ZYHPM01 profile. (a) MCMC iteration count used to generate the gray-scale density plots. (b) Prior (green) and posterior (gray) distributions of the sedimentation rate parameter. (c) Prior (green) and posterior (gray) distributions of the autocorrelation parameter, reflecting the vertical dependency of accumulation rates between adjacent depths. (d) Calibrated OSL ages (purple; 2σ uncertainty) overlain on the age–depth model. Dark gray shading indicates the most likely calendar ages; light gray dashed lines mark the 95% confidence intervals. The red curve denotes the weighted-mean age trajectory.

The chronological framework was established using five OSL samples (Table 2). Analysis of the De distributions reveals distinct characteristics across the profile. Samples from 0.2, 0.9, and 1.1 m exhibit concentrated De distributions with low overdispersion (OD), indicating well-bleached sediments; the Central Age Model (CAM) was applied to these. Samples from 0.6 and 1.5 m display broader, multimodal dose distributions with higher OD. For these, the Finite Mixture Model (FMM) was employed to isolate the primary depositional component. The resulting ages range from 4.80 ± 0.65 ka to 11.63 ± 1.25 ka and follow a consistent stratigraphic order with no reversals. A Bayesian age-depth model was then constructed using the Bacon package [30] in R, incorporating all five OSL ages and their uncertainties to generate a continuous chronostratigraphic framework with 95% credible intervals (Fig 3).

**Table 2:** Optically stimulated luminescence dating results of the ZYHPM01 profile.

Sample No	Profile depth	Grain size	U	Th	K	Equivalent dose	Environmental Dose Rate	Water content	Age Model	Age
	(m)	μm	(μg/g)	(μg/g)	(%)	(Gy)	(Gy/ka)	(%)		(ka)
ZYHPM01–0.2	0.2	125-180	2.86 ± 0.1	11.7 ± 0.51	1.73 ± 0.02	12.60 ± 1.69	2.63 ± 0.09	29	CAM	4.80 ± 0.65
ZYHPM01–0.6	0.6	125-180	3.16 ± 0.14	13 ± 0.45	1.77 ± 0.01	14.83 ± 2.25	2.90 ± 0.10	23	FMM	5.11 ± 0.77
ZYHPM01–0.9	0.9	125-180	3.23 ± 0.16	12.8 ± 0.43	1.82 ± 0.02	19.44 ± 0.71	2.90 ± 0.10	25	CAM	6.72 ± 0.25
ZYHPM01–1.1	1.1	125-180	2.96 ± 0.12	12.7 ± 0.6	1.92 ± 0.03	28.42 ± 0.98	2.88 ± 0.09	28	CAM	9.86 ± 0.35
ZYHPM01–1.5	1.5	125-180	2.59 ± 0.08	11.8 ± 0.49	2.06 ± 0.01	32.80 ± 3.54	2.82 ± 0.10	28	FMM	11.63 ± 1.25

The Bayesian age-depth model reveals substantial variations in sedimentation rate throughout the Holocene, with an overall mean of 13.3 cm/ka. Based on the age-depth relationship and sedimentological characteristics, the depositional history is divided into five stages. Stage I (1.6–1.2 m, 12.07–10.31 ka BP) records rapid accumulation at ~22.7 cm/ka. Stage II (1.2–0.9 m, 10.31–6.72 ka BP) is characterized by markedly reduced sedimentation (~8.4 cm/ka). Stage III (0.9–0.6 m, 6.72–5.11 ka BP) shows a recovery in accumulation rate to ~18.6 cm/ka. Stage IV (0.6–0.4 m, 5.11–4.96 ka BP) records an abrupt surge in deposition; however, the calculated rate (~133 cm/ka) should be interpreted with caution owing to the large OSL age uncertainties relative to the short time interval. The sedimentary record shows enhanced deposition and coarser grain-size composition during this interval, but the exact magnitude of the rate increase is poorly constrained. Stage V (0.4–0 m, 4.96 ka BP to near-present) is characterized by a relatively low sedimentation rate of 8.1 cm/ka. It should be noted that only the lower part of this stage (0.4–0.2 m) is directly bracketed by OSL ages; ages for the uppermost 0.2 m are extrapolated from the Bayesian age-depth model and carry correspondingly wider uncertainty (Fig 3; see also Section 4.1).

### 3.3. Grain size characteristics

**Fig 4:**
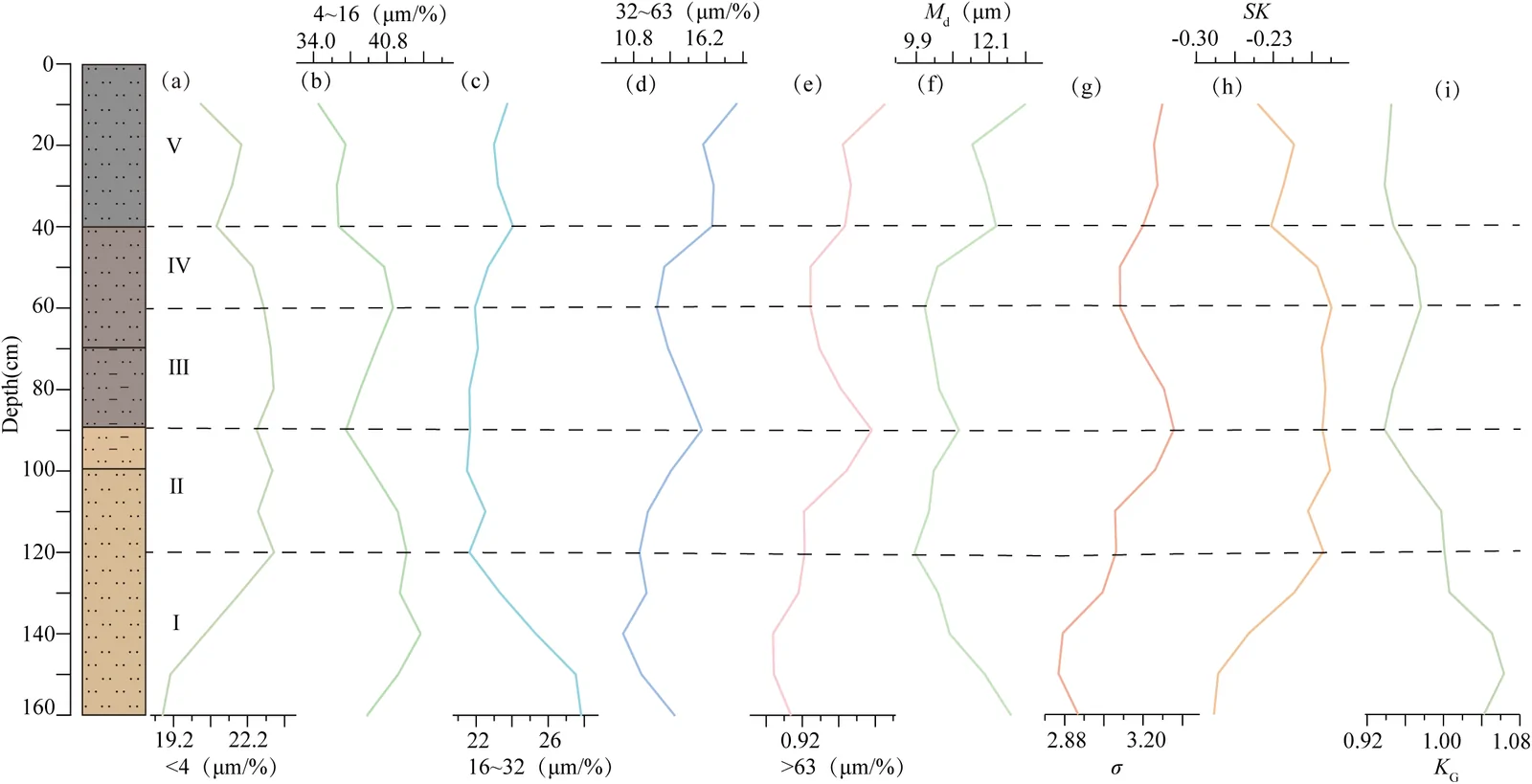
Down-profile variations in grain-size parameters. From left to right: clay (<4 µm), fine silt (4–16 µm), medium silt (16–32 µm), coarse silt (32–63 µm), sand (>63 µm), median grain size (*M*_d_), sorting coefficient (*σ*), skewness (SK), and kurtosis (K_*G*_).

**Fig 5:**
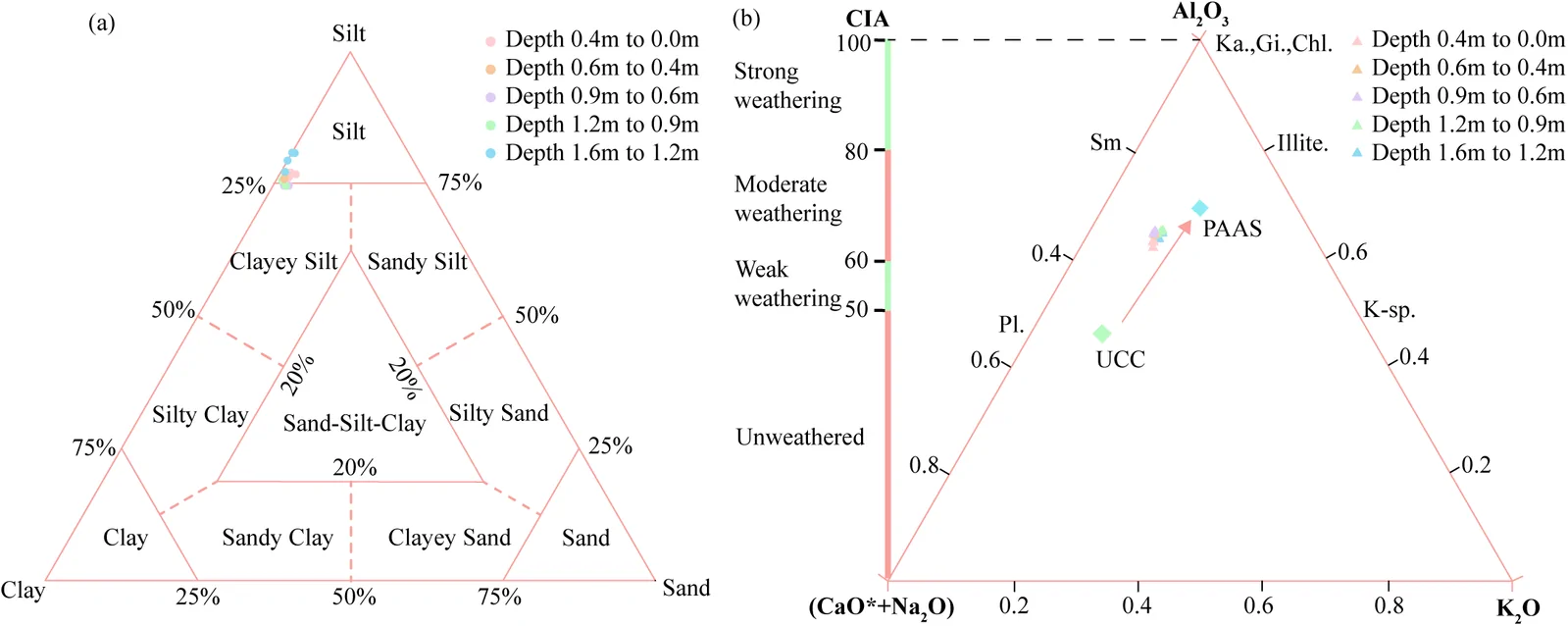
Ternary diagrams for sediment classification and weathering assessment. (a) Shepard triangular diagram showing the grain-size classification of the samples. (b) A-CN-K (Al_2_O_3_–(CaO* + Na_2_O)–K_2_O) triangular diagram showing the weathering trend of the samples relative to the UCC and PAAS, where CIA denotes the Chemical Index of Alteration. The CIA value increases from the UCC corner toward the Al_2_O_3_ apex. Predicted weathering trajectories for plagioclase and K-feldspar are shown as arrows.

The ZYHPM01 sediments are predominantly silt and clayey silt with low sand content (Table 3, Fig 4). On the Shepard ternary diagram (Fig 5a), all samples cluster within the silt and clayey silt fields. Fine silt (4–16 µm) is the dominant fraction (avg. 39.47%), followed by medium silt (16–32 µm; avg. 23.35%), clay (<4 µm; avg. 21.94%), and sand (>63 µm; avg. 1.44%). The vertical variation in grain-size parameters defines five stages (Fig 4). Stage I (1.6–1.2 m) shows a fining-upward trend, with median grain size (Md) decreasing from 12.77 to 9.84 µm and clay content increasing from 18.74% to 23.54%. Stages II and III (1.2–0.6 m) constitute the finest interval, with Md fluctuating narrowly between 9.84 and 11.19 µm and clay content averaging ~23%. A sharp textural discontinuity marks Stage IV (0.6–0.4 m), where Md increases abruptly from 10.53 to 12.31 µm. Stage V (0.4–0 m) contains the coarsest deposits (peak Md = 13.21 µm; sand up to 3.01%).

**Table 3:** Summary of contents and parameters of sediment grain size fractions.

Sample No	depth	<4 μm	4 ~ 16 μm	16 ~ 32 μm	32 ~ 63 μm	>63 μm	M_*d*_	σ	SK	KG
	(m)	(%)	(%)	(%)	(%)	(%)	(μm)			
L1	0.1	20.38	34.46	23.72	18.42	3.01	13.21	3.28	−0.24	0.95
L2	0.2	22.12	36.98	23.00	15.95	1.95	11.59	3.24	−0.21	0.94
L3	0.3	21.73	36.17	23.21	16.73	2.15	12.01	3.26	−0.22	0.94
L4	0.4	21.05	36.31	24.05	16.59	2.00	12.31	3.20	−0.23	0.95
L5	0.5	22.61	40.53	22.66	13.08	1.12	10.53	3.11	−0.18	0.97
L6	0.6	23.10	41.35	21.93	12.51	1.12	10.15	3.11	−0.17	0.98
L7	0.7	23.39	39.80	22.11	13.35	1.35	10.38	3.19	−0.18	0.96
L8	0.8	23.53	38.33	21.63	14.60	1.90	10.59	3.28	−0.18	0.95
L9	0.9	22.80	37.00	21.67	15.85	2.68	11.19	3.32	−0.18	0.94
L10	1	23.47	39.42	21.50	13.58	2.04	10.43	3.25	−0.17	0.97
L11	1.1	22.85	41.80	22.52	11.87	0.96	10.28	3.09	−0.19	1.00
L12	1.2	23.54	42.63	21.62	11.22	0.98	9.84	3.09	−0.18	1.00
L13	1.3	22.09	41.99	23.33	11.76	0.83	10.56	3.03	−0.21	1.01
L14	1.4	20.57	43.90	25.33	10.03	0.18	10.91	2.87	−0.25	1.05
L15	1.5	19.06	41.82	27.54	11.38	0.20	11.96	2.85	−0.28	1.06
L16	1.6	18.74	38.96	27.84	13.83	0.63	12.77	2.94	−0.29	1.04

**Fig 6:**
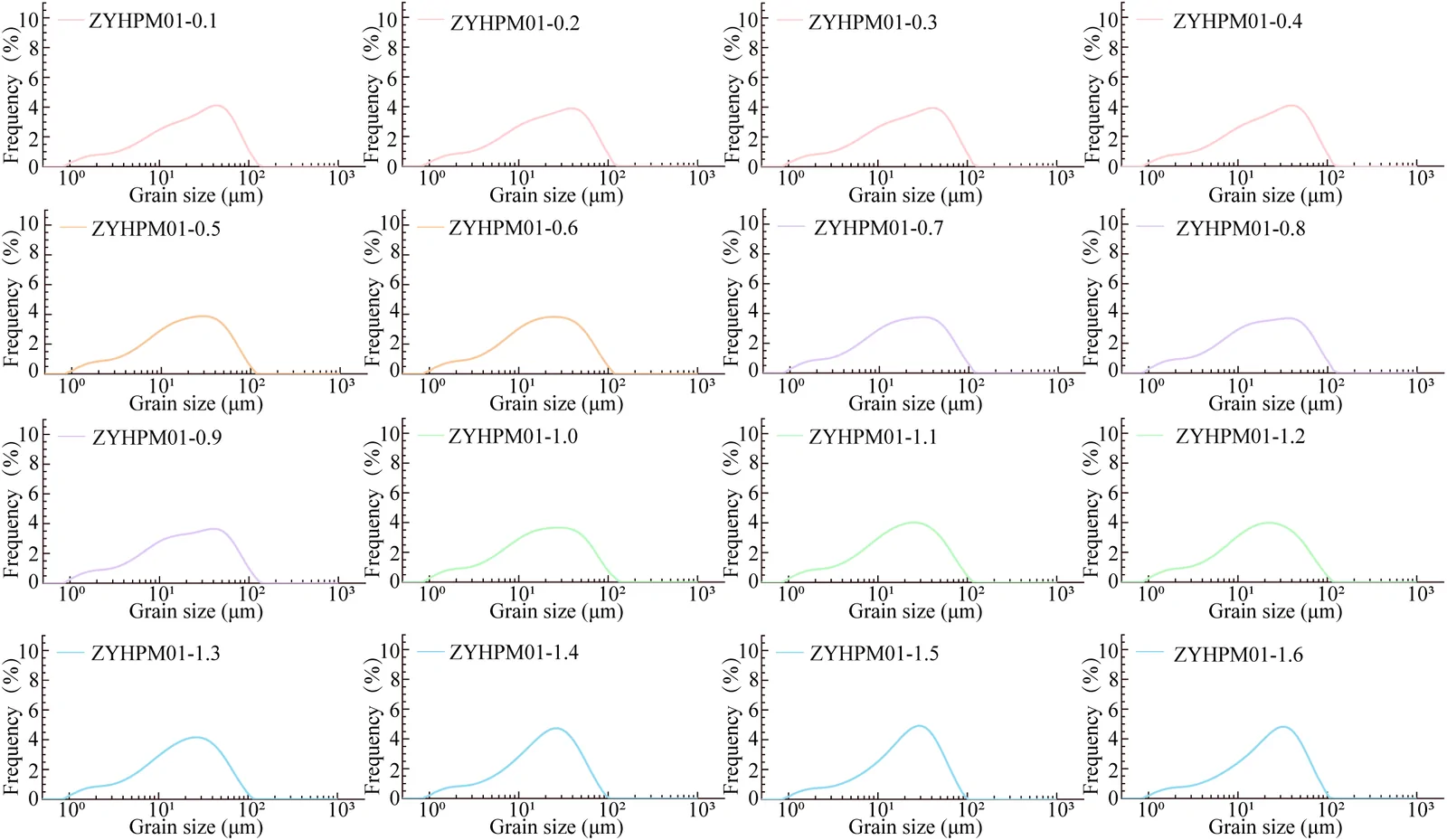
Grain-size frequency distribution curves of the ZYHPM01 profile. Samples are arranged by depth from top to bottom. Curves in Stage I (1.2–1.6 m), light blue curves display a bimodal distribution with a primary coarse-silt peak and a secondary fine-silt peak; the overall fining-upward trend is reflected by a progressive narrowing of the primary peak and an increasing clay-fine silt fraction. Stage II (0.9–1.2 m), light green curves retain the bimodal pattern but with a more symmetrical morphology and a subdued primary peak, corresponding to the finest interval of the profile. Stage III (0.6–0.9 m), light purple curves maintain bimodality yet show a broadening of the coarse-silt peak and an enhanced coarse tail. Stage IV (0.4–0.6 m), orange curves mark a transitional morphology from bimodal to trimodal, with a nascent fine-sand shoulder emerging on the coarse flank. Stage V (0–0.4 m), light red curves exhibit a well-developed trimodal distribution, characterized by a prominent coarse-silt peak, a fine-silt peak, and a distinct fine-sand population.

Statistical parameters support this stage-based division. Sorting (σ) ranges from 2.85 to 3.32, indicating poor sorting throughout. Skewness (SK) values fall between −0.29 and −0.17, with finer layers tending toward more negative values and coarser layers toward less negative values. Kurtosis (K_*G*_) remains near mesokurtic (0.94–1.06). Frequency distribution curves (Fig 6) transition from bimodal in Stages I–III, with a primary coarse-silt peak, to trimodal in Stages IV–V, where an additional fine-sand population emerges.

### 3.4. Grain size end-member modeling

**Fig 7:**
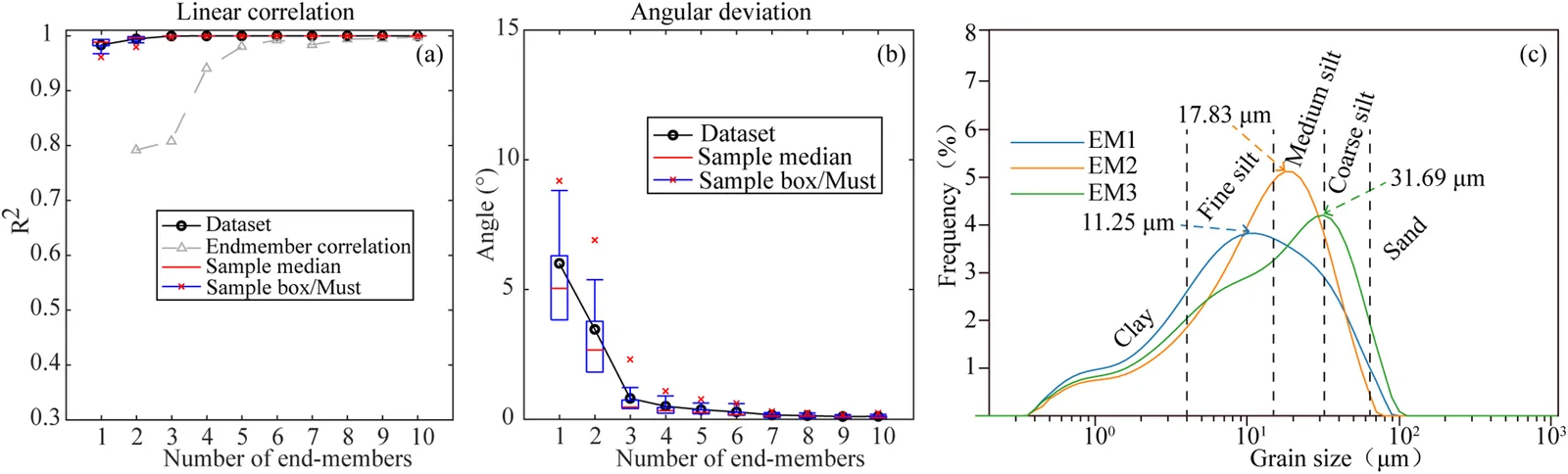
End-member modeling of grain-size data. (a) Linear correlation (R^2^) of the grain-size dataset as a function of the number of end-members. (b) Angular deviation (θ) as a function of the number of end-members. (c) Grain-size distribution curves of the three resolved end-members: EM1 (modal ~11.25 μm, fine silt), EM2 (modal ~17.83 μm, medium silt), and EM3 (modal ~31.69 μm, coarse silt).

Grain-size end-member modeling resolved the dataset into three components, explaining > 95% of the total variance (R^2^ > 0.95, θ < 4.5°; Figs 7a and 7b). EM1 (modal size 11.25 µm, fine silt) is the finest and best-sorted component, with a secondary peak in the clay fraction (<4 µm), and a fine-skewed distribution (Fig 7c). EM2 (modal size 17.83 µm, medium silt) is intermediate in both size and sorting. EM3 (modal size 31.69 µm, coarse silt) is the coarsest and most poorly sorted, with a broad, asymmetrical distribution tailing toward the coarse end (Fig 7c).

**Fig 8:**
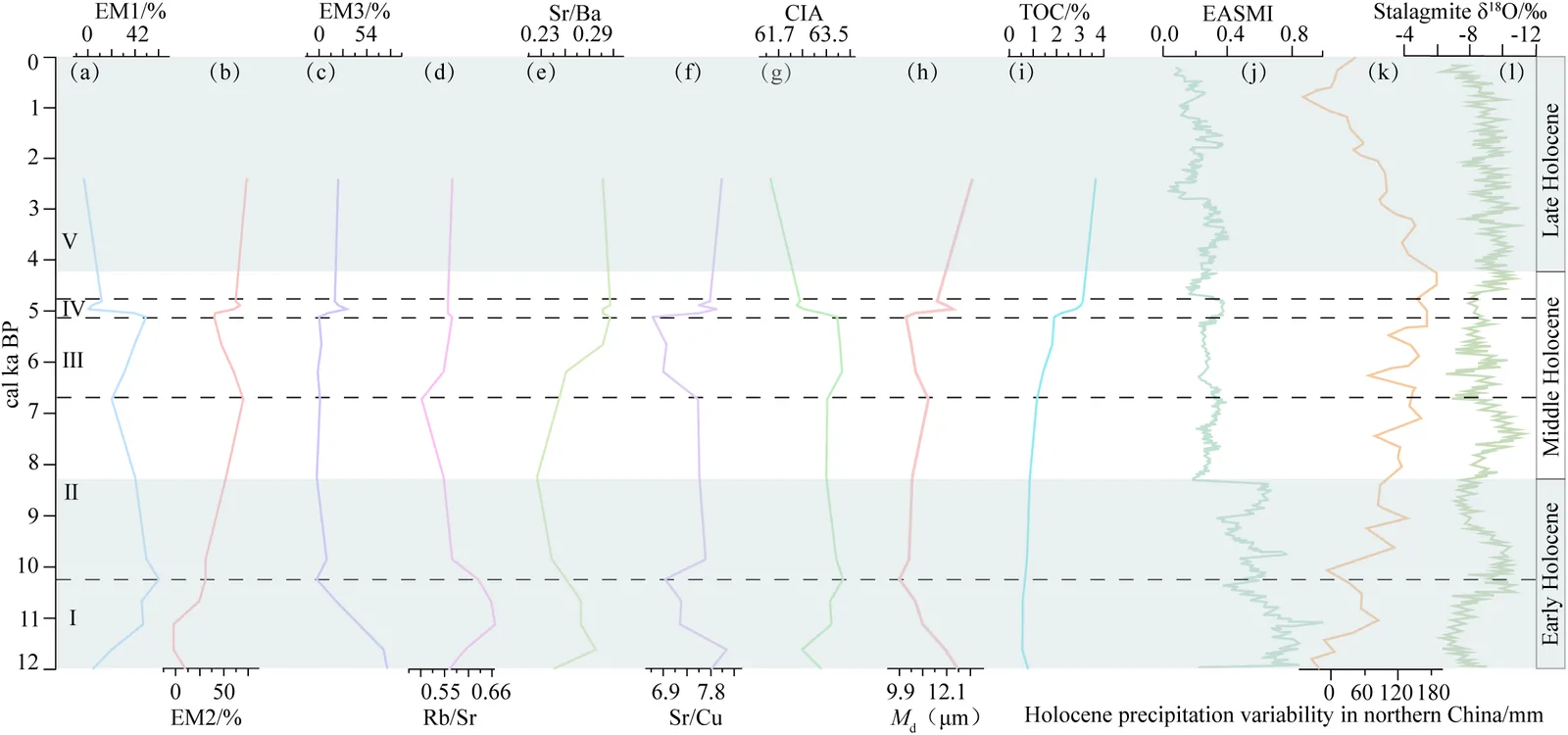
Down-profile variations in grain-size end-members, geochemical proxies, and regional climate records. (a) EM1 proportion, (b) EM2 proportion, (c) EM3 proportion, (d) Rb/Sr ratio, (e) Sr/Ba ratio, (f) Sr/Cu ratio, (g) Chemical Index of Alteration (CIA), (h) median grain size (*M*_d_), (i) Total organic carbon (TOC), (j) East Asian Summer Monsoon (EASM) intensity index [38], (k) Holocene precipitation variability in Northern China [39], (l) δ^18^O of Chinese cave stalagmite calcite from Sanbao Cave [40]. The vertical axis represents age (ka BP) based on the Bayesian age-depth model. Stage boundaries are indicated by dashed lines. Alternating background shading denotes the Early-, Middle-, and Late-Holocene subdivisions.

The stratigraphic variations in EM contributions define the same five-stage structure identified from grain-size parameters (Fig 8). In Stages I–III (1.6–0.6 m), EM1 dominates; within Stage I, its proportion increases upward, paralleling the fining-upward trend. A significant compositional shift occurs at the Stage IV boundary (0.6 m), marked by a sharp decline in EM1 and a concurrent surge in EM3. In Stage V (0.4–0 m), the coarser components EM2 and EM3 reach their maximum cumulative abundance, whereas EM1 drops to its profile minimum.

### 3.5. Geochemical characteristics

**Fig 9:**
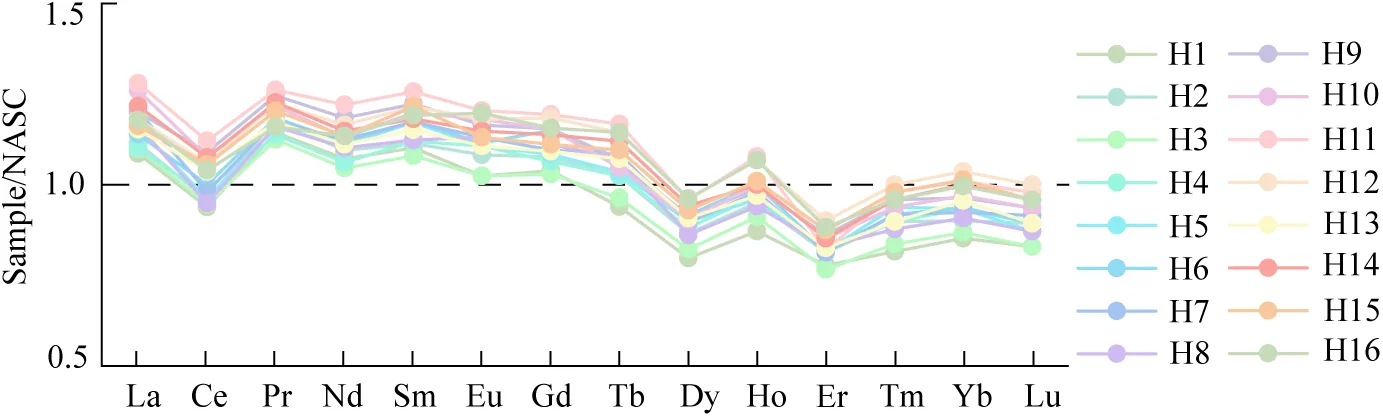
Chondrite-normalized rare earth element (REE) patterns of the ZYHPM01 samples. Normalization values are from Taylor and McLennan [41]. Coloured curves represent the range of all 16 samples.

The bulk geochemistry of the ZYHPM01 profile (Table 4) is dominated by SiO_2_ (59.45%–63.30%, avg. 61.11%) and Al_2_O_3_ (13.54%–15.13%, avg. 14.47%), followed by TFe_2_O_3_ (4.96%–7.22%, avg. 5.95%). Mobile elements are relatively depleted, with CaO and Na_2_O averaging 1.51% and 1.81%, respectively. On the A-CN-K ternary diagram (Fig 5b), all samples plot above the Post-Archean Australian Shale (PAAS) line, deviating from the Upper Continental Crust (UCC) toward the Al_2_O_3_ apex. Trace element ratios (e.g., La/Sc, Th/Sc) and chondrite-normalized REE patterns (Fig 9, Table 5) are remarkably uniform throughout the profile, characterized by slight LREE enrichment and negative Eu anomalies.

**Table 4:** Summary of sediment element contents and parameters. Element contents of the upper continental crust are from Taylor and McLennan [41].

Sample No	SiO_2_	Al_2_O_3_	TFe_2_O_3_	MgO	CaO	Na_2_O	K_2_O	TOC	Ba	Cu	Rb	Sc	Sr	Th	CIA	La/Sc	Th/Sc	Rb/Sr	Sr/Ba	Sr/Cu
	(%)	(%)	(%)	(%)	(%)	(%)	(%)	(%)	(μg/g)	(μg/g)	(μg/g)	(μg/g)	(μg/g)	(μg/g)						
L1	60.53	13.54	4.96	1.38	1.72	1.76	2.32	3.70	597.00	23.40	113.00	12.90	192.00	11.70	61.99	2.69	0.91	0.59	0.32	8.21
L2	61.00	13.97	5.36	1.40	1.72	1.74	2.27	3.15	587.00	24.20	112.00	13.40	193.00	12.10	63.06	2.66	0.90	0.58	0.33	7.98
L3	60.67	13.85	5.41	1.37	1.64	1.74	2.24	3.07	584.00	24.70	112.00	12.80	192.00	11.40	62.96	2.74	0.89	0.58	0.33	7.77
L4	61.15	13.93	5.52	1.36	1.60	1.73	2.23	2.85	609.00	23.80	111.00	12.60	193.00	11.70	63.22	2.79	0.93	0.58	0.32	8.11
L5	62.13	14.17	5.62	1.40	1.48	1.78	2.21	2.25	576.00	24.10	109.00	13.80	187.00	12.40	63.88	2.68	0.90	0.58	0.32	7.76
L6	61.36	14.51	5.82	1.48	1.49	1.79	2.18	1.95	574.00	27.20	110.00	14.70	187.00	12.60	64.42	2.48	0.86	0.59	0.33	6.88
L7	63.30	15.13	5.94	1.48	1.59	1.80	2.28	1.86	602.00	26.60	110.00	14.40	190.00	12.30	64.52	2.67	0.85	0.58	0.32	7.14
L8	61.14	14.95	6.37	1.47	1.57	1.77	2.24	1.48	716.00	27.10	109.00	14.30	192.00	11.50	64.60	2.59	0.80	0.57	0.27	7.08
L9	59.89	14.64	6.61	1.50	1.56	1.82	2.20	1.21	785.00	26.70	107.00	14.50	207.00	13.20	64.07	2.64	0.91	0.52	0.26	7.75
L10	59.54	14.44	7.20	1.49	1.46	1.82	2.28	0.91	853.00	25.20	111.00	14.40	196.00	12.60	64.02	2.79	0.88	0.57	0.23	7.78
L11	60.09	14.57	7.22	1.49	1.40	1.79	2.37	0.78	798.00	24.80	116.00	14.40	196.00	13.50	64.40	2.83	0.94	0.59	0.25	7.90
L12	61.25	15.13	5.89	1.59	1.36	1.89	2.49	0.70	700.00	26.70	124.00	15.30	190.00	13.80	64.64	2.46	0.90	0.65	0.27	7.12
L13	61.92	14.88	5.59	1.57	1.35	1.90	2.52	0.61	649.00	24.90	125.00	14.80	185.00	12.40	64.17	2.49	0.84	0.68	0.29	7.43
L14	62.35	14.90	5.45	1.67	1.36	1.86	2.56	0.60	641.00	25.00	127.00	15.60	185.00	13.20	64.21	2.49	0.85	0.69	0.29	7.40
L15	61.92	14.45	5.84	1.64	1.36	1.96	2.53	0.58	640.00	23.70	123.00	14.90	197.00	12.80	63.14	2.49	0.86	0.62	0.31	8.31
L16	59.45	14.40	7.19	1.46	1.47	1.77	2.29	0.94	845.00	24.80	111.00	14.00	196.00	13.10	64.11	2.69	0.94	0.57	0.23	7.90
UCC	66.60	15.40	5.04	2.48	3.59	3.27	2.80	—	628.00	28	84.00	14.00	320.00	10.50	—	—	—	—	—	—

**Table 5:** Summary of sediment rare earth element contents. Rare element contents of the upper continental crust are from Taylor and McLennan [41], PAAS are from Nance and Taylor [42], and NASC are from Haskin et al. [43].

Sample No	La	Ce	Pr	Nd	Sm	Eu	Gd	Tb	Dy	Ho	Er	Tm	Yb	Lu
	μg/g	μg/g	μg/g	μg/g	μg/g	μg/g	μg/g	μg/g	μg/g	μg/g	μg/g	μg/g	μg/g	μg/g
L1	34.7	68.6	9.02	33.2	6.27	1.27	5.41	0.8	4.65	0.91	2.66	0.41	2.65	0.4
L2	35.6	70.1	9.29	33.9	6.34	1.34	5.6	0.87	5.04	0.99	2.78	0.45	2.8	0.43
L3	35.1	69.2	8.87	32.4	6.15	1.27	5.36	0.82	4.79	0.95	2.62	0.42	2.7	0.4
L4	35.2	70.8	8.97	32.9	6.37	1.37	5.54	0.87	5.13	1.01	2.81	0.45	2.9	0.43
L5	37	70.3	9.13	34.4	6.66	1.37	5.64	0.88	5.22	1	2.79	0.47	2.9	0.42
L6	36.4	73	9.33	34.6	6.68	1.38	5.98	0.9	5.33	1.04	2.79	0.46	2.91	0.43
L7	38.4	71.6	9.26	34.8	6.67	1.4	5.7	0.92	5.21	1.02	2.78	0.46	2.87	0.44
L8	37.1	69.4	9.15	34.1	6.39	1.4	5.78	0.91	5.02	0.98	2.83	0.44	2.82	0.42
L9	38.3	79.4	9.81	36.6	6.95	1.44	6	0.91	5.3	1.03	2.94	0.48	2.99	0.45
L10	40.2	78.5	9.59	35	6.81	1.46	5.99	0.89	5.26	1.04	2.91	0.47	3.01	0.45
L11	40.8	81.7	9.93	37.7	7.14	1.49	6.2	0.99	5.57	1.12	2.85	0.49	3.13	0.47
L12	37.7	77.2	9.64	36	6.91	1.46	6.15	0.97	5.59	1.11	3.07	0.5	3.21	0.48
L13	36.8	75.6	9.24	34.4	6.55	1.37	5.68	0.91	5.28	1.01	2.82	0.45	2.97	0.43
L14	38.8	78.4	9.66	35.5	6.72	1.42	5.92	0.95	5.47	1.04	2.91	0.48	3.1	0.46
L15	37.1	77	9.48	35.1	6.91	1.4	5.78	0.93	5.4	1.05	2.98	0.49	3.14	0.46
L16	37.6	75.9	9.14	35.1	6.77	1.48	6.01	0.97	5.58	1.11	3.01	0.48	3.09	0.46
UCC	31	63	7.1	27	4.7	1	4	0.7	3.9	0.83	2.3	0.3	1.96	0.31
PAAS	38	79.6	8.83	33.9	5.55	1.08	4.66	0.77	4.68	0.99	2.85	0.41	2.82	0.43
NASC	32	73	7.9	31	5.7	1.24	5.21	0.85	5.8	1.04	3.4	0.5	3.1	0.48

Weathering and environmental proxies (CIA, Rb/Sr, Sr/Ba, Sr/Cu, and TOC) vary systematically across the five stages (Fig 8). In Stage I (1.6–1.2 m), the CIA fluctuates between 63.14 (at 1.5 m) and 64.64 (at 1.2 m), showing an overall weak increasing trend from the base upward, albeit with an excursion to lower values at 1.5 m, while Rb/Sr shows a convex trend, peaking at 0.69 at 1.4 m; Sr/Cu declines gradually upward. Stage II (1.2–0.9 m) records a synchronous decline in CIA, Rb/Sr, and Sr/Ba to local minima, accompanied by increasing Sr/Cu. In Stage III (0.9–0.6 m), CIA rebounds and stabilizes at the highest stage-mean value in the profile (avg. 64.40), Rb/Sr and Sr/Ba recover, and Sr/Cu reaches its lowest levels (avg. ~ 7.3); TOC increases markedly from 1.21% at 0.9 m to 1.95% at 0.6 m; however, the highest TOC values in the profile occur in Stage V. Stage IV (0.6–0.4 m) marks a sharp inflection: Sr/Cu increases rapidly, CIA drops abruptly to ~63.2, whereas TOC continues to rise. In Stage V (0.4–0 m), Sr/Cu increases further, and CIA declines to its profile minimum (62.0); TOC remains elevated, particularly in the surface layer.

## 4. Discussion

### 4.1. Reliability of chronology and stability of provenance

**Fig 10:**
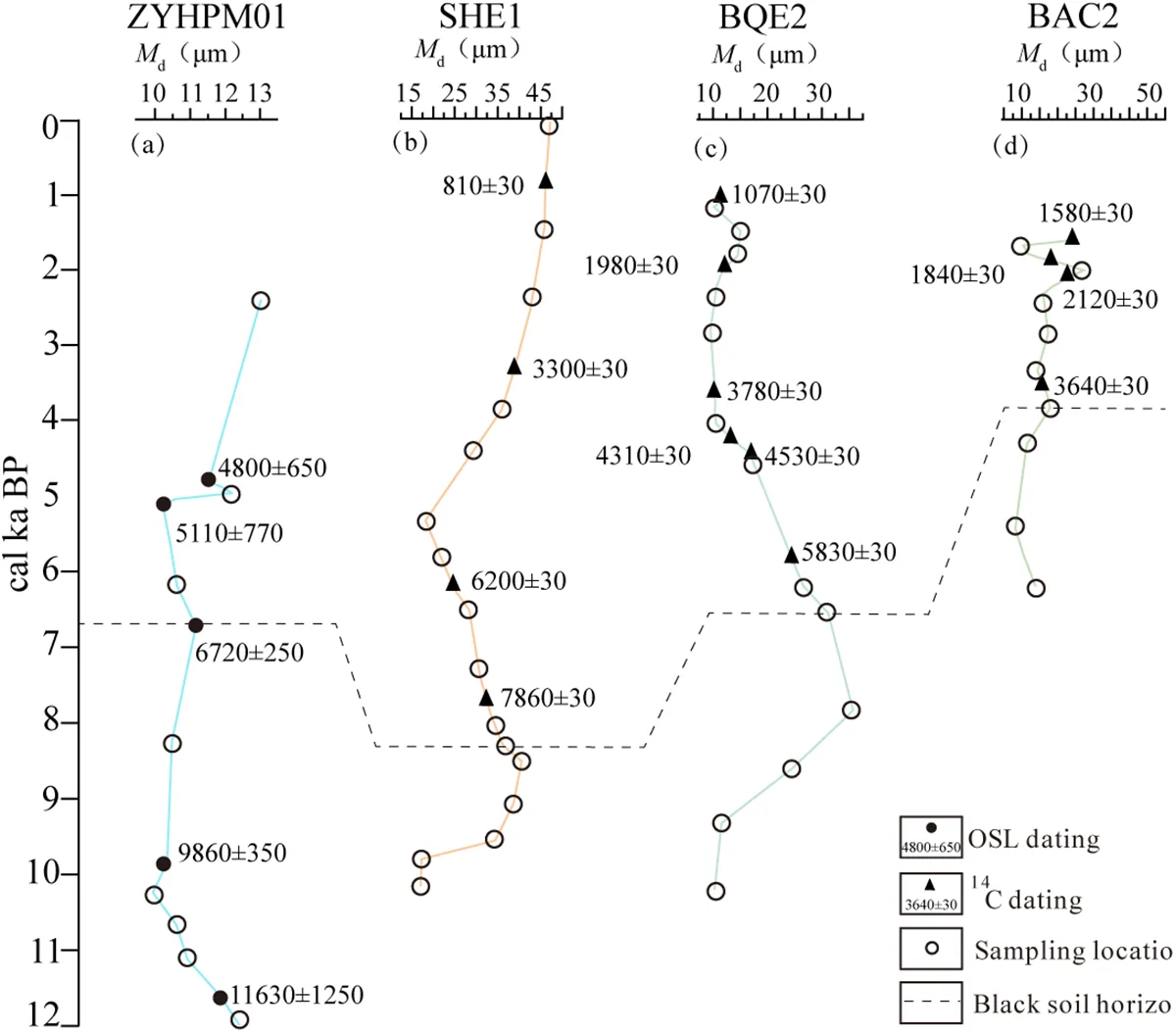
Comparison of median grain size (Md) records from Holocene profiles in the eastern Songnen Plain. The ZYHPM01 profile (this study) is compared with profiles SHE1, BQE2, and BAC2 [9]. The horizontal axis represents age (cal ka BP). Dashed horizontal lines denote the approximate stratigraphic range of the black soil horizon in each profile. Note the consistent regional pattern of mid-Holocene fining (high clay and fine silt content during ~6.5–5.0 ka) followed by late-Holocene coarsening, supporting the climatic interpretation of the ZYHPM01 record.

Paleoenvironmental reconstruction from soil profiles requires a reliable chronology and the exclusion of provenance interference. In black soils, this is complicated by pedoturbation, which can mix soil particles and blur depositional signals [8]. Several lines of evidence support the reliability of our chronological framework. First, coarse-grained quartz (125–180 µm) was used for OSL dating; unlike fine silt (4–11 µm), this fraction is resistant to vertical migration via illuviation or bioturbation, thereby preserving the primary stratigraphic order [44,45]. Second, for samples with broad or multimodal De distributions (0.6 and 1.5 m), the FMM was applied to isolate the primary depositional population from intrusive grains [46]. Third, the resulting ages (4.80–11.63 ka) follow a consistent stratigraphic order with no reversals, and the preservation of sharp lithological boundaries, particularly the abrupt coarsening at the Stage III/IV transition, provides supporting physical evidence that post-depositional homogenization was limited. Fourth, the Bayesian age-depth model explicitly incorporates dating uncertainties, yielding continuous 95% credible intervals that strengthen the chronological confidence. Comparative analysis with regional records further validates the chronology (Fig 10). One additional consideration concerns the uppermost part of the profile. The top 0.2 m lies above the youngest OSL sample (0.2 m, 4.80 ± 0.65 ka), so its age is constrained only by extrapolation of the Bayesian age-depth model, and it has additionally been disturbed by cultivation. We therefore do not equate the profile top with the present day, and we refer to the upper boundary of Stage V as “near-present” throughout.

Although raw depth-series grain-size records across the Songnen Plain appear spatially heterogeneous, isochronous alignment reveals a coherent regional signal: the ~ 6.5 ka horizon marks both the widespread onset of black soil formation [7,9] and a critical granulometric turning point across multiple profiles. The temporal evolution of grain size in our profile, mid-Holocene fining followed by late-Holocene coarsening, aligns well with high-resolution regional records, indicating that the ZYHPM01 profile records broad-scale climatic evolution rather than localized geomorphic noise. Regarding provenance, the geochemical data indicate a stable source throughout the Holocene. The chondrite-normalized REE patterns (Fig 9) are uniform, and diagnostic trace-element ratios (La/Sc, Th/Sc) vary within narrow ranges consistent with the UCC [41,47]. This homogeneity indicates that the observed variations in grain-size and weathering proxies reflect climatic dynamics rather than shifts in source lithology.

### 4.2. Paleoenvironmental significance of sedimentary proxies and weathering indices

Interpretation of the environmental significance of each proxy is necessary before reconstructing the Holocene evolution. In the following discussion, the proxies are organized along a conceptual chain linking monsoon-modulated dust supply (grain-size end-members), chemical weathering intensity (CIA and Rb–Sr systematics), and pedogenic carbon accumulation (TOC). The three grain-size EMs are interpreted as distinct transport modes on the basis of their modal grain sizes, analogy with published end-member signatures, and regional geomorphological evidence. EM1 (modal ~11.25 µm, fine silt) closely matches the fine-grained background component resolved in end-member models of the Harbin loess [48] and the grain-size range of far-travelled dust recorded by modern dust-fall monitoring [49]; it is therefore interpreted as background dust transported via high-altitude suspension, analogous to typical Chinese loess [50,51]. EM2 (modal ~17.83 µm, medium silt) falls within the grain-size range of reactivated dune sands and near-source dust reported from the Horqin and Songnen sandy lands [16,17,52] and is interpreted as regional dust from such proximal sources supplied during periods of dune activation under a strengthened winter monsoon [53]. EM3 (modal ~31.69 µm, coarse silt) is interpreted as proximal high-energy saltation or localized surface reworking; its broad, coarse-tailing distribution is also compatible with a contribution from snowmelt- or rainfall-driven wash [54–57], and this alternative interpretation cannot be excluded. We note that end-member modeling is a statistical decomposition of grain-size distributions; the assignment of specific transport mechanisms to individual EMs therefore relies on the analogies outlined above rather than on direct sediment-trapping observations [23,34].

Among the geochemical proxies, the CIA tracks silicate weathering intensity, specifically the transformation of feldspar to clay minerals [36,58]. The Rb/Sr ratio reflects the competition between Sr leaching and Rb retention on clay minerals [47,59]. The Sr/Ba ratio is sensitive to weathering degree. Sr is preferentially leached from plagioclase under humid conditions, whereas Ba is more stable and retained in K-feldspar and clay minerals, so lower Sr/Ba indicates more intense weathering. The Sr/Cu ratio indicates the balance between physical erosion and chemical weathering. Sr is abundant in fresh detrital plagioclase but depleted by leaching, whereas Cu is retained in clay and organic complexes [60,61], thus, low Sr/Cu signifies enhanced leaching under warm-humid conditions, and elevated Sr/Cu indicates enhanced physical erosion under cold-dry conditions [62]. TOC reflects the net balance between biomass production and preservation, the latter controlled by burial rate, microbial decomposition, and temperature [63,64].

### 4.3. Holocene climate evolution and environmental drivers

Integration of chronology, grain-size, and geochemical records reveals five stages of environmental evolution in the eastern Songnen Plain, driven by the interplay between the East Asian summer and winter monsoons. Following the conceptual chain outlined in Section 4.2, each stage is characterized in terms of sediment supply (EM assemblage and sedimentation rate), weathering intensity (CIA, Rb/Sr, Sr/Ba, and Sr/Cu), and carbon accumulation (TOC), so that the climatic interpretation of each stage is presented once, in integrated form. The multi-proxy characteristics of the five stages and their environmental interpretation are summarized in Table 6.

**Table 6:** Summary of multi-proxy characteristics and environmental interpretation of the five evolutionary stages of the ZYHPM01 profile.

Stage	Age range (ka BP)	Sedimentation rate (cm/ka)	Dominant EM(s)	CIA	Sr/Cu	TOC (%)	Environmental interpretation
I	12.07–10.31	~22.7	EM1 (increasing upward)	63.14–64.64	7.12–8.31	0.58–0.94	Post-glacial warming–wetting; rapid accumulation exceeding pedogenic alteration
II	10.31–6.72	~8.4	EM1 dominant; EM2 increasing	64.02–64.64	7.12–7.90	0.70–1.21	Climatic deterioration during the early-to-middle Holocene transition
III	6.72–5.11	~18.6	EM1 dominant	avg. 64.40	~7.3	1.21–1.95	Mid-Holocene Climatic Optimum; Formation Phase of black soil
IV	5.11–4.96	~133	EM3 surge	63.22–63.88	7.76–8.11	2.25–2.85	Abrupt cold–dry shift; rapid burial of organic matter
V	4.96 to near-present	~8.1	EM2 + EM3 maxima	61.99–63.22	7.77–8.21	2.85–3.70	Neoglacial cooling; Preservation Phase with continued root-zone input

Stage I (12.07–10.31 ka BP): Rapid accumulation and incipient weathering. Following the last glaciation, the strengthening EASM brought warmer and wetter conditions to the region [40,65]. The dominance of fine-grained EM1 and the high sedimentation rate (~22.7 cm/ka) indicate that material supply still exceeded pedogenic alteration. The decoupling of CIA and Rb/Sr described in Section 3.5, CIA rising while Rb/Sr peaks then declines, reflects a shift from transport-limited to weathering-limited conditions: initial Rb adsorption on fine clays gave way to preferential Sr leaching as precipitation intensified [36]. A comparable early Holocene transition from rapid aeolian accumulation to incipient weathering has been documented in the Hulun Buir sandy land [66] and the Horqin sandy land [52]. This stage established the hydrothermal foundation for subsequent soil development.

Stage II (10.31–6.72 ka BP): Weakened weathering during the early-to-middle Holocene transition. The synchronous decline of CIA and Rb/Sr to their profile minima, together with increased EM2 input, marks a sustained interval of weakened chemical weathering and strengthened winter monsoon. Although the sampling resolution (~700 years between adjacent samples) precludes precise identification of short-term events, the broad trend is consistent with early-to-middle Holocene climatic deterioration. The 8.2 ka BP cooling event [67,68] may have contributed to this deterioration, but the ~ 3,600-year duration of Stage II far exceeds the ~ 160-year timescale of that event; the observed trends more likely reflect the integrated response to early Holocene orbital forcing rather than any single abrupt event.

Stage III (6.72–5.11 ka BP): Mid-Holocene climatic optimum and black soil formation. This interval corresponds to the Holocene Climatic Optimum [69], when peak EASM intensity [70] promoted conditions favorable for black soil formation [8]. As detailed in Section 3.5, peak CIA values, the lowest Sr/Cu ratios, and the highest TOC content all point to enhanced leaching under warm-humid conditions. Elevated TOC together with moderate weathering suggests that organo-mineral complexes protected organic carbon from mineralization [71]. Regionally, this stage aligns with maximum Holocene warmth documented in stalagmite δ¹⁸O records from Dongge Cave [65]. The modest CIA difference between the Stage I/II boundary (64.64) and the Stage III average (64.40) indicates that CIA alone cannot discriminate the Climatic Optimum signal; however, the convergent behavior of CIA with Sr/Cu, Rb/Sr, and TOC within Stage III supports the interpretation of enhanced chemical weathering during this interval.

Stage IV (5.11–4.96 ka BP): Enhanced sedimentation and environmental transition. A brief but dramatic increase in sedimentation rate (~133 cm/ka), accompanied by sharp increases in EM2 and EM3, marks a major shift in the depositional regime. The concurrent CIA decline and Sr/Cu increase indicate that fresh, less-weathered material overwhelmed the pedogenic signal. The rapid burial likely preserved contemporaneous organic matter from atmospheric oxidation, accounting for the rising TOC trend despite deteriorating weathering conditions. This environmental shift pre-dates the widely recognized ~4.2 ka BP climate event [72] by approximately 800 years; while a direct causal link cannot be established, the Stage IV transition may reflect an early regional expression of the broader mid-late Holocene monsoon decline, consistent with evidence for aridification around 4.2 ka BP in the western Loess Plateau [73]. The extreme sedimentation rate likely reflects localized geomorphic processes, episodic dune activation, surface runoff, or extreme aeolian events, superimposed on the background climatic trend.

Stage V (4.96 ka BP to near-present): Neoglacial cooling and organic carbon preservation. Under persistent winter monsoon dominance, this stage aligns with Neoglacial cooling [74,75]. The resurgence of coarse EM2 and EM3, declining CIA, and stable Sr/Ba indicate that physical sediment supply outpaced chemical alteration. Despite suppressed weathering, TOC continued to rise toward the surface. This decoupling is tentatively attributed to: (1) reduced microbial decomposition under cooling, which inhibited organic matter mineralization more than primary productivity; and (2) continued input from the active root zone in the Mollic epipedon [9]. This TOC–CIA decoupling under deteriorating weathering conditions has also been reported from other black soil profiles in the Songnen Plain [8] and is further discussed in Section 4.4.

### 4.4. Mechanism of black soil formation

The stage-by-stage integration presented above (Table 6) converges on a two-phase model of black soil development. The black soils of the eastern Songnen Plain formed through a multi-source and multi-process system in which accretionary deposition and climatic modulation acted in concert. The continuous influx of fine-grained distal dust (EM1), geochemically comparable to the UCC and typical loess deposits [48,49,76], provided the material substrate with sufficient specific surface area for organic matter adsorption. This background input was dynamically modified by episodic coarser proximal dust (EM2 and EM3) from nearby sand lands during cold-dry intervals [15,39] and, in the case of EM3, by localized surface reworking such as snowmelt runoff [54,55]. Notably, the CIA value at the profile base (1.6 m, 12.07 ka BP) is already 64.11—close to the profile maximum (64.64 at 1.2 m), indicating that CIA alone has limited power to discriminate weathering stages within such a narrow range (61.99–64.64); the convergent behavior of multiple proxies is therefore essential for environmental interpretation.

The transformation of these sediments into mature black soil required a specific environmental window during the Mid-Holocene (Stage III). As documented in Section 3.5, peak CIA values and the lowest Sr/Cu ratios indicate moderate chemical weathering under warm-humid conditions [36,58]. The decomposition of primary minerals and the formation of secondary clays, combined with high biological productivity, promoted stable organo-mineral complexes that sequester soil organic carbon [71]. The Mid-Holocene Climatic Optimum thus represents the critical interval when moderate chemical weathering, enhanced biomass production, and active pedogenesis combined to establish the distinctive properties of the black soil.

Regarding the timing of formation, multi-proxy records indicate that mature black soil development was initiated at ~6.72 ka BP, consistent with the accretionary pedogenesis model [7,8], which posits that black soils thicken upwards synchronously with Holocene dust deposition. While Cui et al. [9] proposed an earlier onset (no later than 8.5 ka BP) based on ^14^C dating, our data suggest a distinction: although sediment accumulation began earlier (Stages I and II), the distinctive physicochemical properties of the black soil (e.g., peak clay retention, and TOC accumulation) were only fully established during the Climatic Optimum. The material body of the soil is thus time-transgressive, whereas its pedogenic identity is a product of the Climatic Optimum.

The persistence of this organic-rich layer is consistent with a formation-preservation coupling, which we propose as a working hypothesis rather than a directly demonstrated process. Peak chemical weathering and biomass production occurred during Stage III (Formation Phase), whereas maximum TOC retention is recorded in Stages IV-V (Preservation Phase). The subsequent environmental deterioration inhibited microbial respiration and restricted chemical leaching [9], effectively preserving the organic carbon accumulated during the Formation Phase. However, the continued rise of TOC in Stages IV and V, despite declining CIA, cannot be explained by preservation alone; additional contributions from rapid burial (Stage IV) and active root-zone input in the Mollic epipedon (Stage V) are also required [9]. The inference that cooling suppressed microbial decomposition more than primary productivity remains indirect, as microbiological or biochemical constraints are lacking; this limitation is discussed further in Section 4.5.

This interpretation is consistent with independent evidence for carbon preservation in Northeast China. Low mean annual temperatures and a long frozen season suppress microbial mineralization of soil organic matter in the region [77], while the high clay and fine-silt contents documented here favor the formation of stable organo-mineral complexes that protect organic carbon from decomposition [71,78]. At the regional scale, comparable Late Holocene TOC enrichment under deteriorating weathering conditions has been reported from other Mollisol profiles in the Songnen Plain [8,9], and regional soil organic carbon inventories are consistent with the persistence of thick organic-rich horizons in the black soil region [3]. These convergent lines of evidence support—although they do not directly demonstrate—the proposed preservation mechanism. Direct tests of this hypothesis will require microbiological or biomarker-based constraints on decomposition rates.

### 4.5. Limitations of the research

Several limitations should be acknowledged. First, the findings derive from a single sediment profile, which constrains the ability to capture spatial variability across the Songnen Plain; multi-profile studies are needed to assess regional heterogeneity. Second, the 10 cm sampling interval (average ~700 years between adjacent samples) is adequate for millennial-scale trends but insufficient to resolve centennial events such as the 8.2 ka cooling episode. Third, the chronology rests on only five OSL dates. The uppermost age (4.80 ± 0.65 ka) carries a relatively large uncertainty (~13.5% relative error), and the Bayesian age-depth model accordingly assigns wider confidence intervals to the upper portion of the profile. In particular, the uppermost 0.2 m is not constrained by any direct date and may additionally have been homogenized by cultivation; interpretations involving this interval are therefore framed in terms of multi-proxy patterns rather than precise ages. Future work should incorporate independent dating methods (e.g., AMS ¹⁴C) to cross-validate the OSL chronology. Fourth, while the CIA is a robust weathering indicator, it can be influenced by sediment provenance and source lithology; however, the stable La/Sc and Th/Sc ratios throughout the profile argue for a consistent source, supporting the interpretation of CIA variations as primarily weathering-driven. Finally, the proposed formation–preservation coupling mechanism is inferred from geochemical proxies alone; direct microbiological or biochemical evidence is unavailable, and the relative contributions of burial effects, root-zone input, and temperature-controlled preservation to Late Holocene TOC dynamics remain unconstrained.

## 5. Conclusions

This study reconstructs the Holocene evolution of black soil in the eastern Songnen Plain using OSL-based Bayesian chronostratigraphy, grain-size end-member modeling, and multi-proxy geochemical analysis. The principal findings are:

(1) The Bayesian OSL chronology establishes a robust Holocene stratigraphic framework for the ZYHPM01 profile, revealing five evolutionary stages with highly variable sedimentation rates (8.1–133 cm/ka).
(2) Grain-size end-member modeling identifies a dynamic mixture of distal background dust (EM1) and episodic proximal aeolian inputs (EM2 and EM3), with their relative contributions modulated by the East Asian monsoon system.
(3) The Mid-Holocene Climatic Optimum (6.72–5.11 ka BP) provided the primary environmental window for moderate chemical weathering and black soil formation; however, the persistence of the organic-rich layer is consistent with a Formation–Preservation coupling, whereby Mid-Holocene productivity was subsequently preserved by Late Holocene climatic cooling. The multi-proxy records are thus consistent with the hypothesis proposed in the introduction, although direct microbiological constraints remain to be obtained.
(4) These findings highlight the sensitivity of black soil resources to monsoon-driven sedimentary dynamics and imply that future warming may enhance microbial decomposition and chemical leaching, potentially threatening the carbon stock of the Songnen Plain critical zone.
